# Does renal function decline across multiple hospital admissions? An audit of adolescent and young adults hospitalised with a restrictive eating disorder

**DOI:** 10.1186/s40337-026-01658-y

**Published:** 2026-06-25

**Authors:** Elizabeth Kumiko Parker, Michael R. Kohn, Sophie Villar, Anita Stefoska-Needham

**Affiliations:** 1 Department of Dietetics & Nutrition, Westmead Hospital, Westmead, NSW 2145 Australia; 2 Sydney School of Health Sciences, Faculty of Medicine and Health, The University of Sydney, Sydney, NSW 2006 Australia; 3 Peter Beumont Eating Disorders Service, Sydney Local Health District Mental Health Services, Professor Marie Bashir Centre, Level G, 67-73 Missenden Road, Camperdown, NSW 2050 Australia; 4 Paediatrics & Child Health, School of Medicine, Western Sydney University, Campbelltown, NSW 2560 Australia; 5 Centre for Research Into AdolescentS’ Health (CRASH), Westmead Hospital, Westmead, NSW 2145 Australia; 6 Department of Paediatrics, Campbelltown Hospital, Campbelltown, NSW 2560 Australia; 7 Nutrition and Dietetics Discipline, School of Medical, Indigenous and Health Sciences, Faculty of Science, Medicine and Health, University of Wollongong, Wollongong, NSW 2522 Australia; 8 Department of Dietetics, Liverpool Hospital, Liverpool, NSW 2170 Australia; 9 School of Health Sciences, Faculty of Medicine and Health, University of New South Wales, Sydney, NSW 2052 Australia

**Keywords:** Renal function, eGFR, Eating disorders, Protein, Nutritional rehabilitation

## Abstract

**Background:**

Impaired renal function, as indicated by a decline in estimated glomerular-filtration-rate (eGFR), is inadequately investigated in individuals hospitalised with restrictive eating disorders. This study aims to investigate renal function across multiple admissions, as well as the clinical characteristics of this group.

**Methods:**

A five-year retrospective audit was conducted of adolescents and young adults admitted to a hospital in Sydney, Australia, with a restrictive eating disorder requiring multiple admissions for nutritional rehabilitation. eGFR was calculated from serum creatinine levels inputted into the Chronic Kidney Disease in Children Under 25 (CKiDU25) equation. A generalised linear-mixed-model was used to evaluate associations between eGFR and purging history, medical instability on admission, percentage mean BMI (%mBMI), protein intake in grams/kilogram/day, time from last admission, length-of-stay and admission number.

**Results:**

Analysis included 60 patients over 176 admissions, 93% female, mean age 17.1 ± 1.4 years, admission %mBMI 82.0 ± 9.1. The mean number of admissions was 2.9 ± 2.2, with average length-of-stay 22.7 ± 12.2 days, and discharge %mBMI 92.9 ± 7.4. Impaired renal function (eGFR < 90 ml/min/1.73m^2^) was identified in 36.4% of admissions, and in 2.8% of patients at discharge. Average protein intake increased from 2.2 ± 0.6 g/kg/day on admission to 3.0 ± 0.7 g/kg/day on discharge. Protein intake (9.587, *p* < 0.001), %mBMI (0.461, *p* < 0.001), length-of-stay (0.100, *p* < 0.001), and number of admissions (1.921, *p* < 0.001) was positively associated with eGFR. Lower eGFR levels were seen with medical instability (-3.113, *p* = 0.003) or greater between-readmission times (-0.010, *p* < 0.001). The relationship with purging history was not significant.

**Conclusions:**

Impaired renal function was evident in over one third of adolescent and young adults with restrictive eating disorders requiring multiple admissions and improved with nutritional rehabilitation. Findings of this study indicate that eGFR did not decline during the five-year period of observations in this study, however further research is needed to explore the outcomes of the small subgroup of patients who were discharged from hospital with persisting impaired renal function. Overall, eGFR may be a useful indicator of nutritional status and disease severity in patients hospitalised with restrictive eating disorders.

**Supplementary Information:**

The online version contains supplementary material available at https://doi.org/10.1186/s40337-026-01658-y.

## Abstract (plain language summary)

Impaired kidney function has been described as a potential complication of malnutrition in individuals with restrictive eating disorders, however its prevalence in adolescents and young adults is poorly described. It also remains unknown if kidney function declines over time when this patient group requires multiple hospital admissions. This study aims to investigate kidney function across multiple admissions, as well as the clinical characteristics of the group. Kidney function, as indicated by estimated glomerular filtration rate (eGFR), was calculated using the CKiDU25 equation in this five-year retrospective audit of adolescents and young adults requiring greater than one hospital admission for a restrictive eating disorder. Sixty patients were included in the study, representing a total 176 unique admissions. Impaired kidney function was present in 36.4% of admissions and in 2.8% of patients at discharge. A reduced eGFR may be an indicator of disease severity and nutritional status in patients with eating disorders, however the relationship with purging history was not significant.

**Supplementary Information:**

The online version contains supplementary material available at https://doi.org/10.1186/s40337-026-01658-y.

## Background

Eating disorders (EDs) are serious illnesses associated with significant physical and psychological complications [1]. Disturbances in body image, attitudes and behaviours towards food and eating are prominent features [2]. Adolescent and young adult females are disproportionally affected, with peak onset at 15–19 years of age [3, 4]. In 2023, it was estimated 1.1 million people or 4.45% of Australians were living with an eating disorder [4]. Recent Australian data and international studies confirm that EDs are chronic and relapsing conditions, with inpatient readmissions being common; for example, a 10-year Australian cohort study reported substantial recurrent hospitalisations, [5] and U.S. data indicate 30-day readmission rates of over 14% among paediatric patients [6]. Some reasons for readmission include failure to gain or maintain weight post-discharge, or relapse of severe ED cognitions and behaviours [7]. Readmission rates for EDs also vary across health systems and service models in different countries, reflecting differences in access to specialist care, continuity of treatment, and systemic factors [8].

Restrictive EDs, including Anorexia Nervosa (AN) encompassing both the restrictive and binge-purge subtypes, atypical AN (AAN) and avoidant restrictive food intake disorder (ARFID), are characterised by severe energy restriction with or without overexercising, and can result in significant weight loss or inadequate weight gain during a period of growth [9, 10]. Restriction of food intake may lead to poor nutritional status, and in severe cases, to protein-energy malnutrition [11]. Malnutrition resulting from an ED is associated with several complications in the adolescent and young adult group, including impairments in growth, menses (in females) and bone health [12]. Acute, life-threatening medical instability, characterised by unstable vital signs including bradycardia, hypotension, dehydration, hypothermia, and deranged electrolytes, may also occur [13]. Medical instability caused by protein-energy malnutrition in individuals with EDs requires admission to hospital for nutritional rehabilitation, usually via oral or enteral feeding or a combination [14]. Nutritional rehabilitation, involving a comprehensive approach to improving health through dietary interventions, facilitates medical stabilisation, restoration of nutritional status and body weight, and normalisation of eating behaviours, whilst safely minimising the risk of developing refeeding complications such as refeeding hypophosphatemia and refeeding syndrome [2, 15–17]. In addition to nutritional rehabilitation, input from a multidisciplinary team including medical, nursing, allied health and mental health clinicians is recommended for comprehensive care during an inpatient admission [2, 18–21].

### Impaired renal function in eating disorders

ED-related protein-energy malnutrition may have deleterious impacts on all organ systems of the body, including the renal system [22, 23]. Electrolyte disturbances, nephrolithiasis, alterations in water metabolism, pyuria, proteinuria and haematuria have been reported in the literature [23, 24]. The prevalence of renal impairment in patients with restrictive EDs is variable, depending on the eGFR cut-off employed. Following the Kidney Disease: Improving Global Outcomes (KDIGO) guidelines, [25] eGFR values are used to categorise kidney function. Normal function is defined as eGFR > 90 mL/min/1.73m^2^. eGFR between 60 and 89 mL/min/1.73m2 (Stage 2) may indicate early-stage kidney disease and is often considered a higher threshold for impairment in this population.

Chronic Kidney Disease (CKD) is defined as eGFR < 60 ml/min/1.73m^2^ for at least three months, with or without evidence of kidney damage [25]. Studies applying a higher eGFR threshold (< 90 mL/min/1.73m^2^) have reported a wide prevalence (4%−96%) of renal impairment among hospitalised patients with restrictive EDs [26, 27]. Using the CKD threshold of < 60 ml/min/1.73m^2^, Riva et al. (2021) found that 13% of patients met criteria for more severe renal impairment at admission [28]. Importantly, eGFR is influenced by body composition, with lower body mass index (BMI) associated with reduced estimates, [28] complicating interpretation in EDs characterised by prolonged starvation, such as AN, and significant loss of muscle and fat mass. Structural renal abnormalities, reduced cardiac output, hypotension, and in binge-purge subtypes, electrolyte and acid-base disturbances and dehydration, have all been proposed as contributing factors [13, 22].

Improvements in eGFR during inpatient nutritional rehabilitation have been reported in adolescents and young adults with restrictive EDs, although the magnitude of change varies by population and estimation equation used [29–31]. Despite this, a proportion of patients continue to demonstrate impaired renal function (eGFR < 90mL/min/1.73m^2^) at discharge, with reported rates ranging from 3.2% to 62.3% [29–31]. For example, Downey et al. (2022) observed that just over half of patients presenting with reduced eGFR normalised during admission when using the modified Schwartz equation, although group level improvements were modest [30]. Comparable partial recovery has been reported using the Cockcroft-Gault equation in European cohorts [31]. Data on eGFR post hospital discharge, or across repeated hospital admissions for nutritional rehabilitation, remain limited.

### Measurement of renal function

Accurate measurement of kidney function in this population is challenging. Direct measurement of GFR is impractical in routine care, leading to reliance on serum creatinine-based estimating equations [32] The modified Schwartz equation is widely applied to children and adolescents aged 1–16 years, [33] while the validated Chronic Kidney Disease in Children Under 25 equation (CKiDU25) extends applicability into young adulthood (up to 25 years of age) [34] and demonstrates improved performance through age- and sex-dependent constants [35]. Although the Cockroft-Gault equation has been recommended by some authors for adolescents with EDs, [27] its accuracy in younger, malnourished individuals is inconsistent, [36, 37] particularly in the context of low muscle mass and altered protein intake [22, 23]. At present, no practical, non-invasive reference standard exists for assessing eGFR in this group [23].

Another concern for kidney health, reported by practising clinicians, is whether providing high levels of protein during higher energy nutritional rehabilitation has the potential to further impair patients’ kidneys when they are already in a compromised state. High protein intake can have both acute and chronic effects on kidneys, especially in CKD patients, [38] however, is generally considered safe for those with normal eGFR [39]. A study by Knight et al. (2003) found women with mild renal insufficiency (eGFR 55–80 ml/min/1.73m^2^) following a long-term high protein diet showed significantly faster declines in eGFR [40]. When looking at the ED populations, evidence is scare. While adequate nutrition is essential for reversing protein-energy malnutrition, the absence of ED-specific clinical guidelines for protein prescription during nutritional rehabilitation contributes to uncertainty about optimal protein intake [2, 19–21, 41].

A prior study by the authors (Thompsonet al., 2025) reported improvements in eGFR among adolescents and adults hospitalised with restrictive EDs during nutritional rehabilitation, with protein intake increasing from 1.9 ± 0.4 g/kg/day at admission to 2.7 ± 0.6 g/kg/day at discharge [29]. However, the study was limited to a single admission, [29] and it remains unclear how renal function evolves over time in patients who remained unwell and require subsequent readmissions. The current study aims to extend this work by examining patients with multiple hospital admissions, characterising renal function across admissions using eGFR calculated with the CKiDU25 equation, describing associated clinical characteristics, and evaluating whether renal function declines over time in this cohort. The amount of protein provided during nutritional rehabilitation and its association with eGFR was also explored.

## Methods

### Study design

A retrospective cross-sectional clinical audit was conducted of patients diagnosed with a restrictive ED and admitted to the Adolescent and Young Adult Medicine service at a hospital in metropolitan Sydney, Australia, between January 2016 and December 2020. Eligible patients were aged between 14 and 25 years with multiple admissions for nutritional rehabilitation for a restrictive ED. Patients with a single admission or stays under 48 h were excluded.

The study was guided by the ‘Strengthening the reporting of observational studies in epidemiology’ (STROBE) checklist [42] for cross-sectional studies (Supplementary File 1). This study received ethics approval from the Western Sydney Local Health District Human Research and Ethics committee (HREC/14/WMEAD/18).

### Data collection

Data were obtained from paper-based and electronic medical records and were recorded in Microsoft Excel Version 16.77.1. Baseline data included sex, admission age, height, weight, percentage median BMI (%mBMI), diagnosis, history of purging prior to admission (yes/no), admission number, and medical stability on admission. Medical instability was defined as heart rate < 50 or > 100 beats per minute, temperature < 35 °C, or blood pressure < 80/50mmHg. Serum creatinine levels and test dates were recorded. For each test date, the corresponding weight and details of nutrition support (oral meal plan and/or enteral feed) were recorded. Weight was measured thrice weekly (Monday, Wednesday, Friday), typically aligning with each patient’s blood tests. Missing weights were replaced with the previous weight measurement. There were five standardised meal plan prescriptions, providing between 1800 and 3800 kcal/day and 61–162 g/day protein. If a meal was not finished, patients were provided with a liquid oral nutritional supplement to meet the prescribed energy intake. The nutrition prescription was reviewed at least three times per week by the treating medical team with the aim of progressing total energy intake to achieve weight restoration of a minimum of 1 kg per week, where appropriate. Daily energy (kcal/kg actual weight/day) and protein (g/kg actual weight/day) intake from oral and enteral nutrition prescriptions were calculated. Discharge date, weight, %mBMI and length-of-hospital stay were recorded. Total weight gain during admission was calculated. Percentage mBMI, calculated using the 50th percentile of CDC BMI growth charts, [43] was used to assess malnutrition severity, as it is commonly reported in adolescents and young adults [10].

To give an estimate of GFR in ml/min/1.73m^2^, the CKiDU25 equation was used [34]. Inputs into the CKiDU25 equation included mg/dL of creatinine, sex, height in centimetres, and age in years.

In the present study eGFR less than 90 ml/min/1.73m^2^ was chosen as the cut-off value to define clinically significant impaired renal function, in line with other studies investigating impaired renal function in the EDs population [26–31, 44, 45]. Though the study population did not present with CKD, the degree of renal impairment was further categorised according to the KDIGO guidelines for CKD classification, [25] as follows: Stage 1: ≥ 90 mL/min/1.73m^2^ (normal or high); Stage 2: 60–89 mL/min/1.73m^2^ (mildly decreased); Stage 3 A: 45–59 mL/min/1.73m^2^ (mildly to moderately decreased); Stage 3B: 30–44 mL/min/1.73m^2^ (moderately to severely decreased); Stage 4: 15–29 mL/min/1.73m^2^ (severely decreased); and Stage 5: < 15 mL/min/1.73m^2^ (kidney failure).

### Statistical analysis

Data collected was de-identified and inputted into IBM SPSS Statistics version 28.0 (IBM Corp, 2023) [46] for analysis. Descriptive statistics were used to describe patient demographics and clinical data, including counts (n), range, mean, standard deviation and percentages. Variables reported included length-of-hospital admission stay, weight and %mBMI on admission and discharge, average weight gain per week, and total weight and %mBMI change throughout admission.

A generalised linear mixed model was used to evaluate associations between eGFR, calculated using the CKiDU25 equation, and various dependent variables. The model accounted for fixed effects including purging history, medically unstable on admission, %mBMI, protein intake in g/kg/day, time from last admission, length-of-stay and admission number. An interrupted time series was included in the model and was analysed using segmented regression analysis. The two-time variables included in the model were ‘admission duration’ and ‘number of days between the first day (day 1) of each admission’. These time periods did not overlap, complying with the requirements of the model. Subject number was accounted for as a random effect. Statistical significance was set at p value < 0.05.

## Results

Of the 445 admissions screened during the 5-year study period, 60 individual patients requiring multiple admissions were identified as meeting the inclusion criteria. Of the 197 admissions identified for these 60 individual patients, data was collected for a total of 176 unique admissions. Twenty-one admissions were excluded, for reasons including admission for weight stabilisation (*n* = 14), transfer to another facility (*n* = 4), and admission < 48 h (*n* = 3). Average patient age on admission was 17.1 ± 1.4 years, admission %mBMI was 82 ± 9.1%, average number of admissions was 2.9 ± 2.2, and average length-of-stay was 22.7 ± 12.2 days (Table 1). Of the 60 patients included in the study, 93% were female (*n* = 56).

The most frequent ED diagnosis was AAN (53.4%), followed by AN (42.6%) and ARFID (4.0%). In 48 admissions (27.3%), patients were identified as having a recent history of purging through self-induced vomiting. Medical instability on admission was identified in 84 admissions (47.7%) and five admissions (2.8%) resulted in discharge against medical advice. Additional patient characteristics are presented in Table 1.

**Table 1:** Characteristics of adolescent and young adult admissions with a restrictive eating disorder (60 individual patients across 176 total admissions)

Demographic and clinical characteristics	Mean (SD)	Median (LQ-UQ)
Age on admission (years)	17.7 (1.5)	17.5 (16.7–18.5)
Number of admissions	2.9 (2.2)	2.0 (2.0–3.0)
Length-of-stay (days)	22.7 (12.2)	20.0 (14.0–29.0)
Admission weight (kg)	45.9 (6.3)	45.2 (42.1–49.4)
Discharge weight (kg)	52.0 (5.5)	51.5 (48.2–55.9)
Total weight gain (kg)	6.1 (3.3)	5.5 (3.8–7.9)
Average weight gain per week (kg)	2.1 (1.1)	1.9 (1.5–2.5)
Admission %mBMI	82.0 (9.1)	81.6 (77.4–86.6)
Discharge %mBMI	92.9 (7.4)	93.2 (89.1–96.1)
Total %mBMI change	10.9 (6.1)	9.7 (6.6–13.8)
Maximum creatinine (umol/L)	65.1 (12.4)	64.0 (58–72)
Nutrition Prescription		
Energy intake on admission		
(kcal/day)	2520.9 (551.6)	2400.0 (1890.0–2800.0)
(kcal/kg/day)	55.6 (13.3)	54.5 (45.6–62.3)
Energy intake on discharge		
(kcal/day)	3526.5 (739.5)	3400.0 (3050.0–3850.0.0.0)
(kcal/kg/day)	69.5 (16.6)	70.0 (59.7–78.3)
Protein intake on admission		
(g/day)	100.0 (23.2)	96.0 (79.0–112.6)
(g/kg/day)	2.2 (0.6)	2.1 (1.8–2.4)
Protein intake on discharge		
(g/day)	150.7 (33.1)	148.7 (132.9–167.1)
(g/kg/day)	3.0 (0.7)	3.0 (2.5–3.4)

%mBMI Percent median body mass index

Average energy intake on admission was 2520.9 ± 551.6 kcal/day, equivalent to 55.6 ± 13.3 kcal/kg/day. Average protein intake increased from 2.2 ± 0.6 g/kg/day on admission to 3.0 ± 0.7 g/kg/day on discharge.

Table 2 summarises the number of admissions with normal eGFR and those where eGFR had decreased from normal levels. Using the CKiDU25 equation, 64 (36.4%) admissions had impaired renal function (eGFR < 90 ml/min/1.73m^2^) on admission versus five (2.8%) with impaired renal function (eGFR < 90 ml/min/1.73m^2^) at discharge.

**Table 2:** Number (%) of admissions with normal (Stage 1) or impaired eGFR (Stages 2–5) on admission and discharge using CKiDU25 equation (*n* = 176)

	eGFR on admission	eGFR on discharge
Stage 1: ≥90 ml/min/1.73m^2^	112 (63.6%)	171 (97.2%)
Stage 2: 60–89 ml/min/1.73m^2^	62 (35.2%)	5 (2.8%)
Stage 3 A: 45–59 ml/min/1.73m^2^	2 (1.1%)	0
Stage 3B: 30–44 ml/min/1.73m^2^	0	0
Stage 4: 15–29 mL/min/1.73m^2^	0	0
Stage 5: < 15 mL/min/1.73m^2^	0	0

*eGFR* Estimated glomerular filtration rate; *CKiDU25* Chronic Kidney Disease in Children Under 25 equation

In the generalised linear mixed model, several independent variables were significantly associated with eGFR (Table 3). These variables included medical instability on admission, %mBMI, protein intake (g/kg), time between admissions, length-of-stay and admission number. Estimates represent the change in eGFR (ml/min/1.73m^2^) per unit change in the model term in the respected variable. Notably, increase in protein intake by 1 g per kg of body weight was associated with an eGFR increase of 9.587 ml/min/1.73m^2^ (95% CI 8.4,10.8). Conversely, medical instability on admission and longer intervals (number of days) between admissions were associated with a decrease in eGFR, −3.113 ml/min/1.73m^2^ (95% CI −5.18, −1.05) and − 0.010 ml/min/1.73m^2^ (95% CI −0.02, −0.01) respectively. History of purging was the only variable that did not show a statistically significant correlation with eGFR.

**Table 3:** Correlations between eGFR and independent variables and eGFR as reported by the CKiDU25 equation (*n* = 176)

Variable	Estimate	Std. Error	t value	*p* value	95% confidence interval	
					Lower	Upper
History of purging	0.505	2.6710	0.189	0.850	−4.734	5.744
Medically unstable	−3.113	1.0540	−2.954	0.003	−5.181	−1.046
%mBMI	0.461	0.0596	7.735	< 0.001	0.344	0.578
Protein (g/kg)	9.587	0.6255	15.326	< 0.001	8.360	10.814
Number of days between admissions	− 0.010	0.0028	−3.690	< 0.001	− 0.016	− 0.005
Length of stay	0.100	0.0221	4.520	< 0.001	0.057	0.144
Number of admissions	1.921	0.2178	8.819	< 0.001	1.493	2.348

*%mBMI* Percentage median body mass index

## Discussion

In this Australian cross-sectional study, eGFR and associated clinical characteristics were explored in a cohort of adolescent and young adult patients with a history of multiple hospital admissions for restrictive EDs. Impaired renal function (eGFR < 90 ml/min/1.73m^2^) was observed in 36.4% of admissions, decreasing to 2.8% at discharge. The observed improvement in eGFR during admission occurred alongside nutritional rehabilitation, including a high-protein intake, indicating that impaired renal function did not persist at discharge in most cases. eGFR was positively associated with %mBMI, greater protein intake (g/kg/day), and length-of-stay, and negatively associated with medical instability and longer intervals between admissions.

Challenges with accurately assessing GFR in children and adolescents, as well as in patients of low body size, have been documented in the literature [36, 37]. To our knowledge, this is the first study to apply the recently developed CKiDU25 equation to an underweight ED population across multiple hospital admissions. Renal function in adolescent and young adults with EDs is poorly investigated in clinical practice despite abnormalities in kidney function being common [26–31, 44, 45]. This is partially due to the lack of agreement on the most appropriate method to use in the adolescent and young adult EDs population to evaluate GFR [27, 36, 37]. As outlined in the Background, previous studies have reported variable rates of impaired renal function on admission and partial improvement during hospitalisation in adolescents with restrictive EDs, with findings influenced by the eGFR equation used [29–31]. Consistent with this literature, most patients in our study with reduced eGFR on admission demonstrated improvement during inpatient nutritional rehabilitation. The improvement observed across multiple admissions aligns with earlier research but appears more pronounced in our cohort. In the current study, a larger proportion of patients admitted with impaired renal function showed improved eGFR to > 90 ml/min/1.73m^2^ during the admission (59/64, 92%), compared to the finding of Downey et al. (2022) [30] and Mignot-Bedetti et al. (2018) [31]. Interestingly, this discrepancy cannot be explained by duration of hospital length-of-stay, as the current study was 22.7 ± 12.2 days, compared with 11 ± 5 days in Downey et al. (2022) [30] and 150 ± 14 days in Mignot-Bedetti et al. (2018) [31].

Potential pre-renal contributors to impaired renal function in AN, including hypovolaemia, hypotension, and bradycardia, have been proposed to reduce renal blood flow; however, evidence across a number of studies suggests that impaired renal function does not consistently align with hydration status alone. Previous investigations in hospitalised adolescents with AN have reported a substantial prevalence of reduced eGFR despite indicators of adequate hydration or following rehydration, leading some authors to speculate that mechanisms beyond dehydration may be implicated [26, 30, 44]. A more detailed discussion of these mechanisms and the supporting evidence is provided in our previously published work [29]. In the present study, interpretation is further limited by the absence of urine-based hydration markers.

In the current study, lower eGFR was associated with medical instability (−3.11 ml/min/1.73m^2^, *p* = 0.003), which included bradycardia. This is consistent with prior reporting of more severe bradycardia in patients with impaired renal function, including Downey et al. (2022) (40 ± 6.5 vs. 41.7 ± 5.8 bmp, *p* = 0.05) [30] and Gurevich et al. (2021) (44 ± 11 vs. 56 ± 16 bpm, *p* < 0.001).^26^ Stheneur et al. (2019) similarly demonstrated an association between reduced heart rate and lower eGFR (*p* = 0.03) using the Cockroft-Gault equation [44] In the current study, %mBMI was positively associated with eGFR (0.461 ml/min/1.73m^2^, *p* < 0.001), aligning with Mignot-Bedetti (2018), who also reported positive correlations between admission BMI and renal function (*r* = 0.22, *p* = 0.02) and weight gain (*r* = 0.64, *p* < 0.01) [31]. Associations between lower BMI or %mBMI and renal impairment have been reported by Riva et al. (2021), [28] Stheneur et al. (2019), [44] and Stheneur et al. (2024) [45]. In contrast, Downey et al. (2022) [30] and Gurevich et al. (2021) [26] did not observe associations between admission BMI or %mBMI and eGFR, respectively. Collectively, these findings highlight links between protein-energy malnutrition, circulatory characteristics, and renal function, supporting the clinical relevance of eGFR as an indicator of illness severity [23].

This study identified a positive association between the number of readmissions and eGFR (*p* < 0.001). One potential interpretation is that patients with previous admissions could have been readmitted earlier in the course of clinical deterioration. This is supported by the lower prevalence of medical instability in the current cohort (84/176, 47.7%), compared with the authors’ previous study of first admissions only (130/187, 69.5%) [29]. Similar rates of impaired renal function at admission were reported in the current study and the authors’ previous study reporting of first admissions only [29] (36.4% in multiple admissions vs. 35.3% single admissions). Rates of renal impairment at discharge were likewise comparable across studies (2.8% vs. 3.2%, respectively) [29]. In the current study, longer intervals between admissions were weakly but significantly associated with lower eGFR (−0.010, *p* < 0.001). This suggests that patients readmitted sooner may present earlier in the course of deterioration, potentially before the development of medical instability.

Poorer renal outcomes in populations with active purging behaviours have been previously reported [22, 23]. However, history of purging was not significantly associated with eGFR in the current study, which may reflect minimal purging behaviours during inpatient care under close monitoring and supervision. It is also possible that the frequency of purging may be a more accurate predictor of eGFR rather than history of purging, which should be explored in future research.

High protein intake is central to nutritional rehabilitation, supporting positive protein balance and muscle anabolism [47]. In this study, patients received high protein, higher-energy regimens (average 2.2 ± 0.6 g/kg/day on admission and progressing to 3.0 ± 0.7 g/kg/day by discharge). Over this period, the prevalence of impaired renal function decreased from 36.4% to 2.8%, and protein intake was positively associated with eGFR (9.587 ml/min/1.73m^2^, *p* < 0.001) in the linear-mixed-model. Improvements in eGFR occurred alongside high protein intake, with no observed decline in renal function.

### Strengths and limitations

This study is the first, to our knowledge, to examine the relationship between protein intake during nutritional rehabilitation and eGFR across multiple admissions in an adolescent and young adult ED population. This study has several limitations that should be considered when interpreting the findings. The retrospective design restricted analyses to variables routinely documented during admission, resulting in some missing anthropometric data and the absence of urine measures that could have provided additional insights into hydration status. The use of creatinine-based equations may underestimate the prevalence and persistence of renal dysfunction in underweight adolescents and young adults, and alternative methods for assessing kidney function may yield different results. The cohort was managed on a specialised medical ward with expertise in eating disorders, which may limit the generalisability of findings to settings without similar clinical resources. Finally, the study focused exclusively on renal function during inpatient admission, preventing conclusions about longer-term renal trajectories or outcomes beyond the hospital setting.

Therefore, further research is indicated. A cohort follow-up study evaluating renal function outside of admission and over a longer period would be valuable in providing more support for our findings. Research is also needed to identify clinically meaningful factors contributing to persistently abnormal eGFR in patients with restrictive EDs post nutritional rehabilitation. Prospective studies could clarify whether persistently reduced eGFR in this population is associated with adverse adult outcomes or if it affects long-term ED management. More controlled intervention studies are required to rigorously determine the optimal protein prescription during nutritional rehabilitation in this population.

## Conclusion

This study demonstrates that impaired renal function was evident in over a third of adolescent and young adults with restrictive EDs requiring multiple admissions and it improved with nutritional rehabilitation. Findings of this study indicate that eGFR did not decline during the five-year period of observations in this study, however further research is needed to explore the outcomes of the small subgroup of patients who were discharged from hospital with persisting impaired renal function. Reduced eGFR in patients with restrictive EDs may be an indicator of disease severity and nutrition status, given that medical instability was associated with worse eGFR, and significant improvements occurred with increases in %mBMI following nutritional rehabilitation. A high protein intake providing 2.2 ± 0.6 g/kg/day on admission and increasing to 3.0 ± 0.7 g/kg/day on discharge, occurred concurrently with improvements in eGFR. More research is needed to confirm the underlying pathophysiological mechanism of impaired renal function in the EDs population, and to investigate longer-term impacts of restrictive EDs on renal function.

## Electronic Supplementary Material

Supplementary Material 1. STROBE Checklist

## Data Availability

The datasets used and/or analysed during the current study are available from the corresponding author on reasonable request.

## Abbreviations

eGFR: Estimated glomerular filtration rate
EDs: Eating disorders
AN: Anorexia nervosa
AAN: Atypical anorexia nervosa
ARFID: Avoidant/restrictive food intake disorder
KDIGO: Kidney disease improving global outcomes
CKiDU25: Chronic kidney disease in children under 25 years
CKD: Chronic kidney disease
BMI: Body mass index
RFS: Refeeding syndrome
