# Supplementary material for: Does renal function decline across multiple hospital admissions? An audit of adolescent and young adults hospitalised with a restrictive eating disorder
Source: J Eat Disord. 2026 Jun 25;14:214. doi: 10.1186/s40337-026-01658-y (PMC13560277; doi:10.1186/s40337-026-01658-y)
Supplement: Supplementary file 1 [file 40337_2026_1658_MOESM1_ESM.doc]

Supplementary File 1. STROBE Checklist

STROBE Statement—Checklist of items that should be included in reports of ***cross-sectional studies***

|  | Item No | Recommendation |
| --- | --- | --- |
| **Title and abstract** | 1 | (*a*) Indicate the study’s design with a commonly used term in the title or the abstract  as stated in the Abstract on page 2 and Patients and  Methods on page 4.  as stated in the Abstract on page 2 and Patients and  Methods on page 4.  Retrospective cross-sectional clinical audit identified in abstract pg 3 and methods p 10. |
|  |  | (*b*) Provide in the abstract an informative and balanced summary of what was done and what was found  Abstract included Page 3-4. |
| Introduction | | |
| Background/rationale | 2 | Explain the scientific background and rationale for the investigation being reported  Included in the Background on page 6-9. |
| Objectives | 3 | State specific objectives, including any prespecified hypotheses  Included in the Background on page 9. |
| Methods | | |
| Study design | 4 | Present key elements of study design early in the paper  Included in the Methods on page 10. |
| Setting | 5 | Describe the setting, locations, and relevant dates, including periods of recruitment, exposure, follow-up, and data collection  Included in the Methods on page 10-11. |
| Participants | 6 | (*a*) Give the eligibility criteria, and the sources and methods of selection of participants  Included in the Methods on page 10. |
| Variables | 7 | Clearly define all outcomes, exposures, predictors, potential confounders, and effect modifiers. Give diagnostic criteria, if applicable  Included in the Methods on page 10-11. |
| Data sources/ measurement | 8* | For each variable of interest, give sources of data and details of methods of assessment (measurement). Describe comparability of assessment methods if there is more than one group  Included in the Methods on page 10-12. |
| Bias | 9 | Describe any efforts to address potential sources of bias  Included in the Methods page 12. |
| Study size | 10 | Explain how the study size was arrived at  Included in the Results on page 13. |
| Quantitative variables | 11 | Explain how quantitative variables were handled in the analyses. If applicable, describe which groupings were chosen and why  Included in the Methods on page 12. |
| Statistical methods | 12 | (*a*) Describe all statistical methods, including those used to control for confounding  Included in the Methods on page 12. |
|  |  | (*b*) Describe any methods used to examine subgroups and interactions  Included in the Methods on page 12. |
|  |  | (*c*) Explain how missing data were addressed  N/A |
|  |  | (*d*) If applicable, describe analytical methods taking account of sampling strategy  N/A |
|  |  | (*e*) Describe any sensitivity analyses  N/A |
| Results | | |
| Participants | 13* | (a) Report numbers of individuals at each stage of study—eg numbers potentially eligible, examined for eligibility, confirmed eligible, included in the study, completing follow-up, and analysed  Included in the Results on page 12. |
|  |  | (b) Give reasons for non-participation at each stage  N/A |
|  |  | (c) Consider use of a flow diagram  N/A |
| Descriptive data | 14* | (a) Give characteristics of study participants (eg demographic, clinical, social) and information on exposures and potential confounders  Included in the Results on page 13-14, Table 1. |
|  |  | (b) Indicate number of participants with missing data for each variable of interest  N/A |
| Outcome data | 15* | Report numbers of outcome events or summary measures  Included in the Results on page 13-16 and summarised in Table 1, 2 and 3. |
| Main results | 16 | (*a*) Give unadjusted estimates and, if applicable, confounder-adjusted estimates and their precision (eg, 95% confidence interval). Make clear which confounders were adjusted for and why they were included  Included in the Results on page 16, table 3. |
|  |  | (*b*) Report category boundaries when continuous variables were categorized  Included in the Results on page 15, table 2. |
|  |  | (*c*) If relevant, consider translating estimates of relative risk into absolute risk for a meaningful time period  N/A |
| Other analyses | 17 | Report other analyses done—eg analyses of subgroups and interactions, and sensitivity analyses  Included in the Results on page 16, table 3. |
| Discussion | | |
| Key results | 18 | Summarise key results with reference to study objectives  Included in the Discussion on page 17. |
| Limitations | 19 | Discuss limitations of the study, taking into account sources of potential bias or imprecision. Discuss both direction and magnitude of any potential bias  Included in the Discussion on page 21-22. |
| Interpretation | 20 | Give a cautious overall interpretation of results considering objectives, limitations, multiplicity of analyses, results from similar studies, and other relevant evidence  Included in the Discussion on page 21-22. |
| Generalisability | 21 | Discuss the generalisability (external validity) of the study results  Included in the Discussion on page 21-22. |
| Other information | | |
| Funding | 22 | Give the source of funding and the role of the funders for the present study and, if applicable, for the original study on which the present article is based  No sources of funding to declare in Declarations on page 24. |

*Give information separately for exposed and unexposed groups.

**Note:** An Explanation and Elaboration article discusses each checklist item and gives methodological background and published examples of transparent reporting. The STROBE checklist is best used in conjunction with this article (freely available on the Web sites of PLoS Medicine at http://www.plosmedicine.org/, Annals of Internal Medicine at http://www.annals.org/, and Epidemiology at http://www.epidem.com/). Information on the STROBE Initiative is available at www.strobe-statement.org.
